# Thioredoxin-Interacting Protein Mediates Apoptosis in Early Brain Injury after Subarachnoid Haemorrhage

**DOI:** 10.3390/ijms18040854

**Published:** 2017-04-18

**Authors:** Qing Zhao, Xudong Che, Hongxia Zhang, Guanping Tan, Liu Liu, Dengzhi Jiang, Jun Zhao, Xiang Xiang, Xiaochuan Sun, Zhaohui He

**Affiliations:** Department of Neurosurgery, the First Affiliated Hospital of Chongqing Medical University, 1 Friendship Road, Chongqing 400016, China; zhaoqingfpp0923@163.com (Q.Z.); che.xudong@163.com (X.C.); 13657672452@163.com (H.Z.); tanguanping@126.com (G.T.); drliu13845@163.com (L.L.); jiangdengzhi@126.com (D.J.); 15123325240@163.com (J.Z.); daisuke0620@163.com (X.X.); sunxch@gmail.com (X.S.)

**Keywords:** thioredoxin-interacting protein, subarachnoid haemorrhage, early brain injury, apoptosis, protein kinase RNA-like ER kinase

## Abstract

Early brain injury (EBI) is considered to be the major factor associated with high morbidity and mortality after subarachnoid haemorrhage (SAH). Apoptosis is the major pathological mechanism of EBI, and its pathogenesis has not been fully clarified. Here, we report that thioredoxin-interacting protein (TXNIP), which is induced by protein kinase RNA-like endoplasmic reticulum (ER) kinase (PERK), participates in EBI by promoting apoptosis. By using adult male Sprague-Dawley rats to establish SAH models, as well as Terminal dexynucleotidyl transferase (TdT)-mediated dUTP nick end labeling (TUNEL) staining, immunofluorescence, and western blot, we found that TXNIP expression significantly increased after SAH in comparison to the sham group and peaked at 48 h (up to 3.2-fold). Meanwhile, TXNIP was widely expressed in neurons and colocalized with TUNEL-positive cells in the hippocampus and cortex of SAH rats. After administration of TXNIP inhibitor-resveratrol (60 mg/kg), TXNIP small interfering RNA (siRNA) and the PERK inhibitor GSK2656157, TXNIP expression was significantly reduced, accompanied by an attenuation of apoptosis and prognostic indicators, including SAH grade, neurological deficits, brain water content, and blood-brain barrier (BBB) permeability. Collectively, these results suggest that TXNIP may participate in EBI after SAH by mediating apoptosis. The blockage of TXNIP induced by PERK could be a potential therapeutic strategy for SAH treatment.

## 1. Introduction

Early brain injury (EBI) refers to the direct brain damage that occurs within 72 h after bleeding and is considered to be the major factor of poor prognosis after subarachnoid haemorrhage (SAH) [1]. Previous studies have confirmed that brain cell apoptosis occurs after SAH and that the levels of numerous apoptotic proteins increased during this process [2,3]. Apoptosis has gradually become regarded as the most important pathological event in aggravating brain cell death, blood-brain barrier (BBB) disruption, and brain edema after SAH [1]. Although some anti-apoptotic strategies have been developed in EBI treatment, the mortality and disability of SAH still remain high, indicating that there may be some unknown mechanisms.

Thioredoxin-interacting protein (TXNIP), also named thioredoxin binding protein-2 or vitamin D3 upregulated protein-1, is widely expressed in the nucleus, mitochondria, and cytoplasm of normal tissue cells [4]. TXNIP directly binds to the active cysteine residue of thioredoxin (TRX) under pathological conditions and further disturbs the TRX/apoptosis signal-regulating kinase-1 (ASK-1) inhibitory complex. This disruption leads to ASK-1 release and activation of the downstream cascade of the apoptosis signalling pathway [5]. Meanwhile, previous studies have found that TXNIP participates in ischaemic diseases [6,7]. Apoptosis is important during EBI, but whether or not TXNIP participates in EBI by mediating apoptosis has not been explored. We previously found that silencing the downstream protein of the protein kinase RNA-like endoplasmic reticulum (ER) kinase (PERK) downstream protein could reduce neuronal apoptosis after SAH [3]. Coincidentally, researchers have recently discovered that PERK, a transmembrane protein in the ER, significantly promotes TXNIP expression [8]. According to these observations, we hypothesized that PERK-induced TXNIP expression may be involved in EBI through its pro-apoptotic function. Therefore, we utilized a TXNIP inhibitor, small interfering RNA (siRNA), and PERK-specific inhibitor to suppress the activity of TXNIP or PERK to confirm that the inhibition of PERK-induced TXNIP expression may exert anti-apoptotic and neuroprotective effects after SAH.

## 2. Results

### 2.1. TXNIP Expression Increased Obviously after SAH

**Figure 1:**
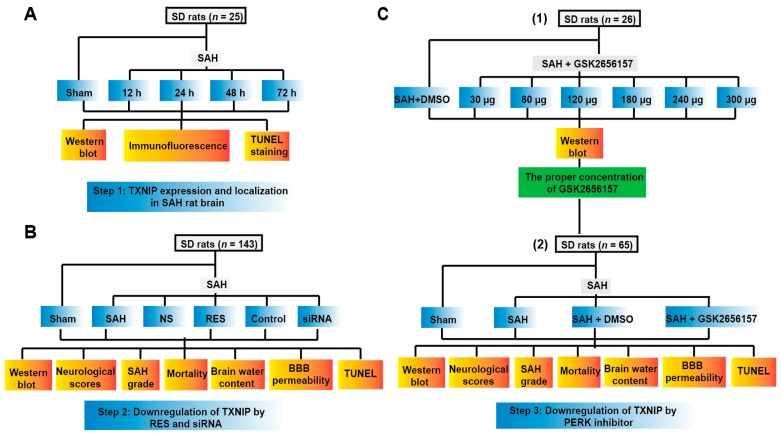
Experimental design (**A**–**C**). SD rats, Sprague–Dawley rats; SAH, subarachnoid haemorrhage; NS, SAH + normal saline; RES, SAH + resveratrol; Control, SAH + control siRNA; siRNA, small interfering RNA; BBB, blood-brain barrier; DMSO, dimethyl sulfoxide; TXNIP, thioredoxin-interacting protein; PERK, protein kinase RNA-like endoplasmic reticulum (ER) kinase.

To confirm our speculation, we designed the experiments shown in Figure 1. Here, rats were divided into different groups, as described in the methods below: sham, SAH, SAH + control siRNA (Control), SAH + TXNIP siRNA, SAH + normal saline (NS), SAH + resveratrol (RES), SAH + dimethyl sulfoxide (DMSO, as a vehicle), and SAH + GSK2656157 (GSK). Both siRNA and GSK2656157 were injected through intracerebroventricular infusion, and RES was administered via intraperitoneal injection.

**Figure 2:**
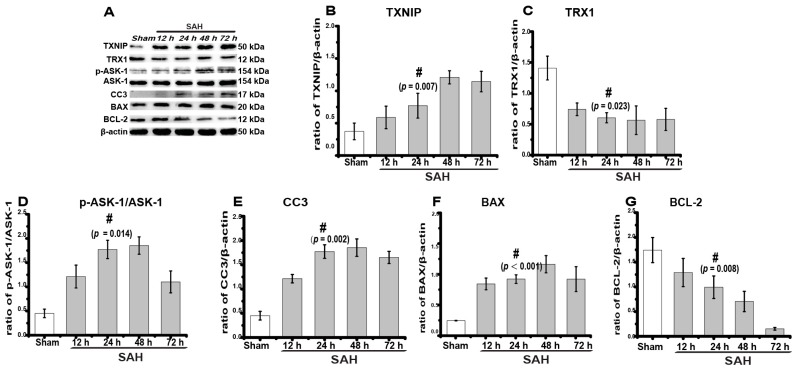
The expression of TXNIP and apoptosis proteins at different time points after SAH. (**A**) Representative western blot of TXNIP, thioredoxin 1 (TRX1), p-ASK-1/ASK-1, cleaved caspase-3 (CC3), B-cell lymphoma (BCL)-2-associated X protein (BAX), and BCL-2; (**B**–**G**) Densitometric quantification of the protein band optical densities for TXNIP and associated proteins. TXNIP expression increased significantly after SAH and was accompanied by an increased expression of apoptosis proteins and decreased expression of anti-apoptotic proteins. # sham vs. SAH at 24 h, *p* < 0.05. ASK-1, apoptosis signal-regulating kinase-1.

First, to detect SAH-induced changes in TXNIP expression and apoptosis, we created SAH models and performed a western blot analysis of the expression of TXNIP, TRX1, p-ASK-1/ASK-1, cleaved caspase-3 (CC3), B-cell lymphoma (BCL)-2-associated X protein (BAX), and BCL-2 at different time intervals (*n* = 6 in each group). Both CC3 and BAX are common apoptosis indexes, while TRX1 and BCL-2 are anti-apoptotic proteins [9,10]. We found that TXNIP expression increased significantly after SAH (Figure 2A,B) at 24 h (0.31 ± 0.02) in comparison to the sham group (0.15 ± 0.05) and peaked at 48 h (0.48 ± 0.04, up to 3.2-fold, *p* = 0.007). The expression of p-ASK-1/ASK-1 (*p* = 0.014), CC3 (*p* = 0.002), and BAX (*p* = 0.000) was also elevated to different extents, accompanied by the downregulation of TRX1 (*p* = 0.023) and BCL-2 (*p* = 0.008) (Figure 2A,C–G). These results indicated that SAH significantly promoted the expression of TXNIP and apoptosis proteins. 

### 2.2. TXNIP Was Expressed in Neurons and Is Colocalized with TUNEL Positive Cells

Based on our previous clinical and experimental observations, clinical symptoms appear and are significant at 24 h in patients and animals after intracranial aneurysm bleeding and SAH. Meanwhile, in our previous study, we detected significant brain cell apoptosis at 24 h and usually remove the brain tissue at 24 h [3,11]. As such, at the beginning of the experimental design, we aimed to observe the results at 24 h in the following experiments, including immunofluorescence, western blot, and apoptosis detection. In addition, we found that changes in TXNIP expression began at 12 h and were obvious at 48 h. At the same time, differences in TXNIP expression at 24 h was also significant when compared with the sham group. Therefore, the results of immunofluorescence, western blot, and apoptosis detection at 24 h may also provide powerful evidence to support research conclusions.

**Figure 3:**
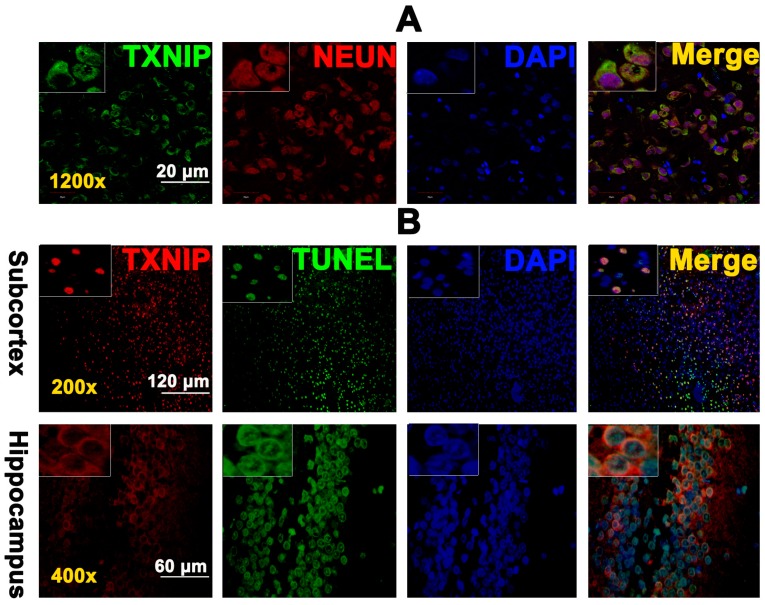
The histological fluorescent analysis of TXNIP and immunofluorescence Terminal dexynucleotidyl transferase (TdT)-mediated dUTP nick end labeling (TUNEL) staining with TXNIP. (**A**) TXNIP was colocalized with NEUN-positive cells (the neurons) (*n* = 3, detected by fluorescence microscopy, FV1200, Olympus, Japan). TXNIP (green), NEUN (red), DAPI (4′,6-diamidino-2-phenylindole, the nucleus, blue), and original magnification ×1200; (**B**) TXNIP was colocalized with TUNEL-positive cells in the hippocampus and subcortex (*n* = 3, detected by fluorescence microscopy, IX71, Olympus, Japan). TXNIP (red), TUNEL (green), DAPI (the nucleus, blue), original magnification of the subcortex (×200) and hippocampus (×400). Scale bars: (**A**) = 20 μm, (**B**) = 120 μm (**top**) and 60 μm (**bottom**).

Because a previous study reported TXNIP expression in neurons of ischaemic–reperfusion brains [12], we also investigated the tissue expression of TXNIP after SAH. Immunofluorescence was performed to detect the localization of TXNIP in the SAH rat brain. Our results showed that TXNIP was widely expressed in the cytoplasm of neurons after SAH (*n* = 3, Figure 3A). To confirm that TXNIP was associated with brain cell apoptosis after SAH, we used Terminal dexynucleotidyl transferase (TdT)-mediated dUTP nick end labeling (TUNEL) staining, a marker for apoptotic cell death [13]. Interestingly, TUNEL analysis demonstrated for the first time that TXNIP was widely colocalized with TUNEL-positive cells in both the hippocampus and cortex of SAH rats (*n* = 3, Figure 3B). These results may provide the critical evidence necessary to support the proapoptotic effect of TXNIP after SAH. 

### 2.3. Downregulation of TXNIP by Resveratrol (RES) and siRNA

**Figure 4:**
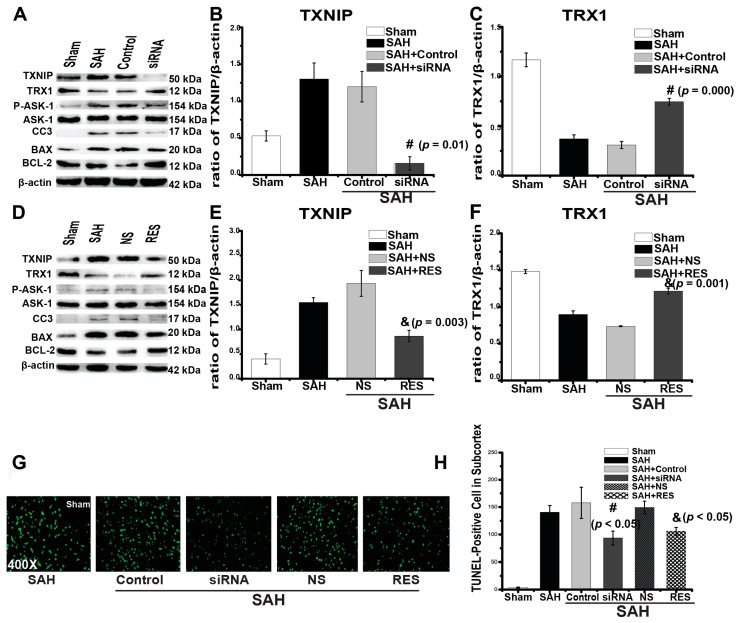
TXNIP and apoptosis protein expression, and the number of TUNEL-positive cells after TXNIP siRNA and RES treatment. (**A**–**F** and Figures S1 and S2) Representative western blot and densitometric quantification of protein band optical densities for TXNIP, TRX1, p-ASK-1/ASK-1, CC3, BAX, and BCL-2. TXNIP and apoptosis protein expression levels were downregulated by TXNIP siRNA and RES treatment; (**G**,**H**) The number of TUNEL-positive cells decreased after siRNA and RES treatment. Original magnification, ×400; scale bars: **G** = 60 μm. # *p* < 0.05 vs. SAH + control siRNA, & *p* < 0.05 vs. SAH + NS.

To further confirm our hypothesis that TXNIP induces apoptosis after SAH, we used RES and TXNIP siRNA to downregulate TXNIP expression. RES is reported to have anti-apoptotic, anti-oxidative, and anti-inflammatory properties [14] and is the most commonly reported strategy to suppress TXNIP and mRNA expression in animal experiments [7,15]. We detected that TXNIP expression increased at 24 h (0.41 ± 0.01) in comparison to the sham group (Figure 4D,E, 0.08 ± 0.01, *p* = 0.001). We found that TXNIP blockage by RES in SAH could significantly reduce the generation of apoptosis proteins (Figure 4D). Meanwhile, RES prevented SAH-induced brain injury and was associated with the reduced expression of TXNIP (*p* = 0.01), p-ASK-1 (*p* = 0.005), CC3 (*p* = 0.000), and BAX (*p* = 0.001) and the increased expression of TRX1 (*p* = 0.000) and BCL-2 (*p* = 0.001) at 24 h after SAH (Figure 4D–F and Figure S2). Brain cell apoptosis was analysed by counting the number of TUNEL-positive cells (*n* = 5), and the number of TUNEL-positive cells in the cortex was reduced by RES injection (*p* < 0.05, Figure 4G,H).

Although TXNIP is a promising therapeutic target, it lacks specific inhibitors and agonists. Additionally, we cannot exclude the possibility that some other mechanisms may also mediate the protection observed with RES treatment, as some researchers have reported that RES may reduce cell apoptosis through the activation of some anti-apoptotic pathways [16]. Thus, we further utilized two previously reported siRNA mixtures to knockdown TXNIP expression [17,18]. We also found that TXNIP expression increased at 24 h (0.31 ± 0.02) in comparison to the sham group (Figure 4A,B, 0.12 ± 0.01, *p* = 0.009). The results showed that TXNIP expression was significantly downregulated by siRNA injection (*p* = 0.003, Figure 4A,B). TXNIP siRNA also decreased the expression of p-ASK-1(*p* = 0.001), CC3 (*p* = 0.001), and BAX (*p* = 0.024), accompanied by an enhanced expression of TRX1 (*p* = 0.001) and BCL-2 (*p* = 0.032) (Figure 4A–C and Figure S1). The number of TUNEL-positive cells was also significantly reduced (*p* < 0.05, Figure 4G,H). Taken together, these results further indicated that TXNIP may aggravate apoptosis after SAH by triggering the downstream apoptosis signalling cascade.

### 2.4. Downregulation of TXNIP by PERK Inhibition

**Figure 5:**
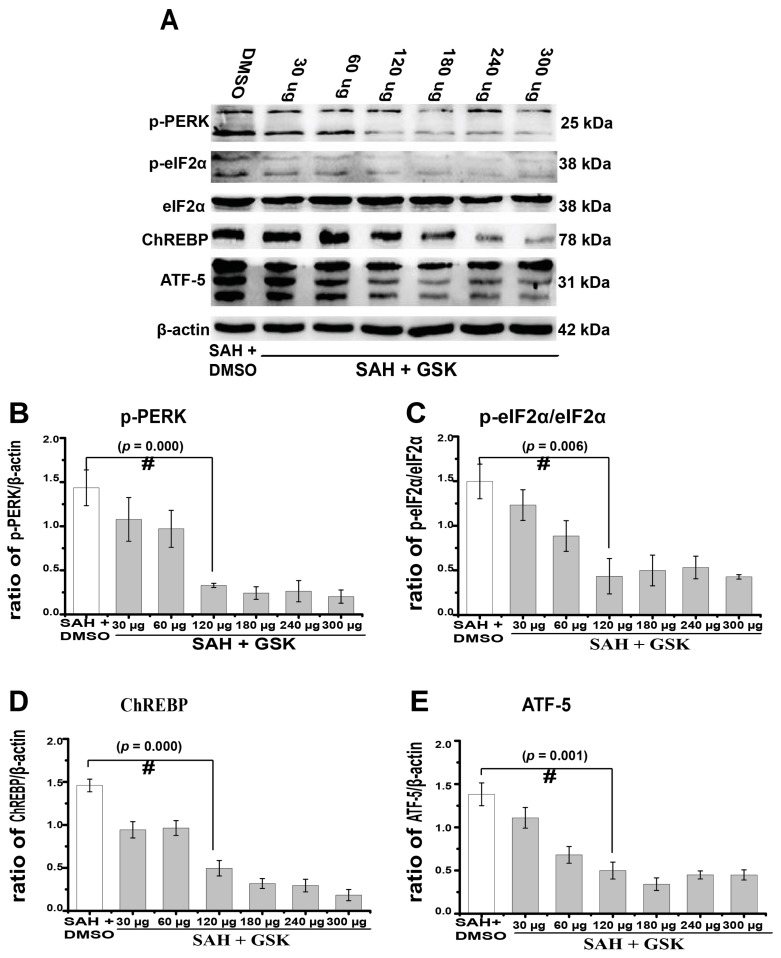
GSK2656157 treatment suppressed the autophosphorylation of PERK in a dose-dependent manner, as well as the expression of p-eIF2α/eIF2α, ATF-5, and ChREBP. (**A**–**E**) Representative western blot of p-PERK, p-eIF2α/eIF2α, ATF-5, and ChREBP. # *p* < 0.05 vs. SAH + DMSO.

**Figure 6:**
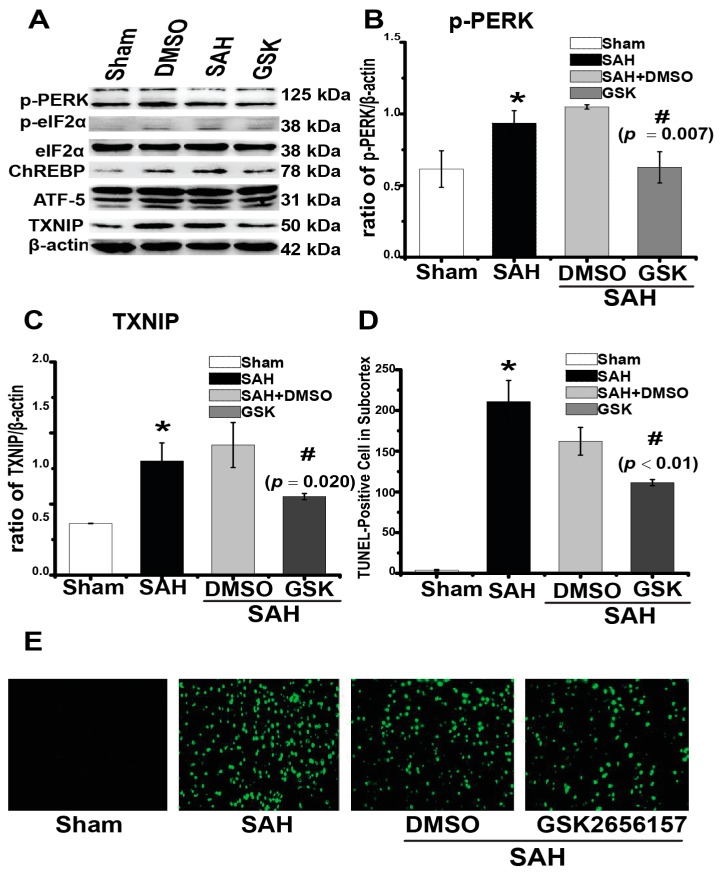
GSK2656157 treatment suppressed TXNIP expression and the number of TUNEL-positive cells. (**A**–**D**, and Figure S3) Representative western blot of p-PERK, TXNIP, p-eIF2α/eIF2α, ATF-5, and ChREBP; (**D**,**E**) The number of TUNEL-positive cells decreased by the GSK2656157 treatment. Original magnification, ×400 (*n* = 5, each group); scale bars: (**E**) = 60 μm. * *p* < 0.05 vs. Sham, # *p* < 0.05 vs. SAH + DMSO.

Simultaneously, we aimed to determine the potential mechanism regulating TXNIP expression after SAH. Our previous work found that silencing the downstream protein of PERK could reduce neuronal apoptosis after SAH [3]. PERK is expressed in the ER transmembrane and has multiple biological functions in the regulation of cell death [19]. More interestingly, researchers recently found that PERK significantly promoted TXNIP expression under ER stress [8]. Thus, we speculated that these mechanisms may also occur after SAH. Therefore, we first inhibited PERK phosphorylation by injecting its specific inhibitor GSK2656157 through intracerebroventricular infusion. We found that PERK phosphorylation was suppressed by GSK2656157 in a dose-dependent manner after SAH, with a significant inhibitory effect from 120 to 300 μg (Figure 5A,B, *p* = 0.000). Meanwhile, the expression of transcription factors downstream of PERK, including phosphorylated eukaryotic translation initiation factor 2α (eIF2α) (*p* = 0.006), activating transcription factor 5 (ATF-5) (*p* = 0.001), and carbohydrate response element binding protein (ChREBP) (*p* = 0.000) transcription factors, was also reduced (Figure 5A,C–E). We then used 300 μg of GSK2656157 for the following experiments, as this dosage showed significant PERK inhibition. TXNIP expression increased at 24 h (0.32 ± 0.01) in comparison to the sham group (Figure 6A,C, 0.10 ± 0.01, *p* = 0.016). TXNIP expression was significantly suppressed by the GSK2656157 (300 μg) treatment (*p* = 0.020, Figure 6A,C). The number of TUNEL-positive cells also decreased by the GSK2656157 treatment (*p* < 0.01, Figure 6D,E). These results indirectly suggested that PERK regulates TXNIP expression after SAH. To some extent, PERK inhibition may downregulate TXNIP expression and reduce apoptosis. We found that the PERK phosphorylation was suppressed by GSK2656157. 

### 2.5. SAH Grade and Mortality

**Figure 7:**
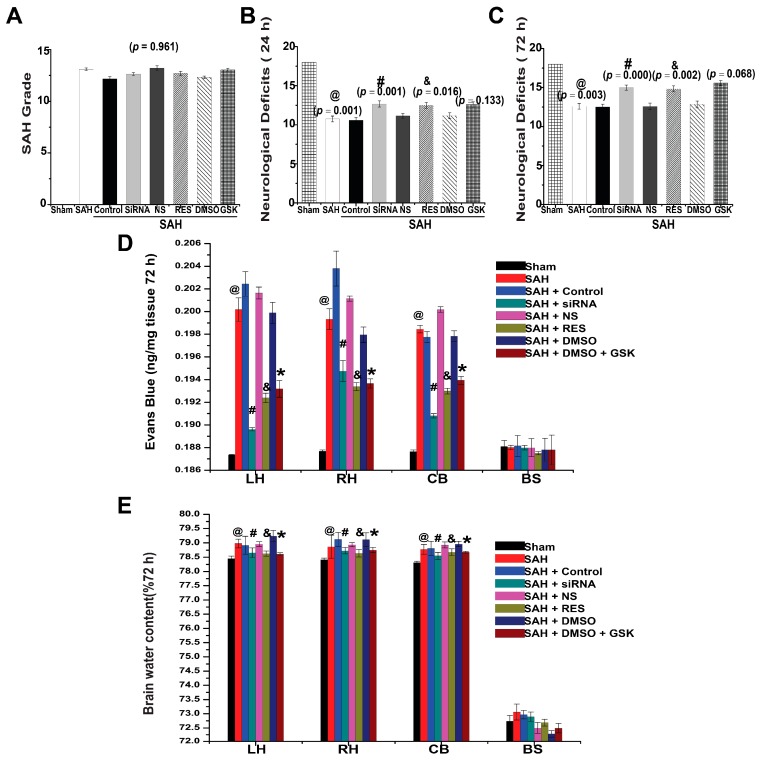
The SAH grade, neurological deficits, blood-brain barrier (BBB) permeability, and brain water content. (**A**) SAH grading scores were similar among each surgery group; (**B**,**C**) TXNIP siRNA and RES increased the neurological score, but GSK2656157 treatment did not result in significant improvement in the neurological deficits compared with the control group; (**D**) BBB disruption was also attenuated in three sections after TXNIP siRNA, RES, and GSK2656157 treatment; (**E** and Table S1) The brain water content was reduced by TXNIP siRNA, RES, and GSK2656157 treatment. @ *p* < 0.05 vs. Sham; # *p* < 0.05 vs. SAH + control siRNA, & *p* < 0.05 vs. SAH + NS, * *p* < 0.05 vs. SAH + DMSO.

Because SAH grade and mortality are prognostic indicators for SAH, they were recorded after surgery and intervention. The SAH grading scores were similar among each surgery group (Figure 7A, *p* = 0.961). A total of 19 rats died during or immediately after operation due to severe SAH, and another 17 rats were excluded because of their low SAH grade. No death was observed in the sham group. Mortality occurred mainly within 24 h after operation. After surgery, 49 rats died within 24 h, and the mortality rates were calculated as follows: 0% (sham, 0 of 33), 23.8% (SAH, 15 of 63), 21.7% (control siRNA, 5 of 23), 14.3% (TXNIP siRNA, 3 of 21), 25.0% (NS, 6 of 24), 18.1% (RES, 4 of 22), 25.0% (DMSO, 7 of 28), and 20.0% (GSK2656157, 9 of 45). However, there were no statistically significant differences in mortality rates between treatment groups after analysis with a Fisher two-sided exact test (*p* = 0.701, control siRNA vs. TXNIP siRNA; *p* = 0.725, NS vs. RES; *p* = 0.772, DMSO vs. GSK2656157).

### 2.6. Neurological Deficits

Neurological scores were measured to evaluate the prognosis of rats 24 h and 72 h after SAH. We found that SAH significantly induced neurological dysfunction after perforation (Figure 7B,C). Compared with the scores of the sham group (18.00 ± 0.00), the neurological scores in the SAH group were significantly lower at 24 h and 72 h (10.75 ± 0.37, *p* = 0.001 and 12.58 ± 0.36, *p* = 0.003, respectively, *n* = 12, Figure 7B,C). In contrast, there were no significant differences among each control group (Figure 7B,C, *p >* 0.05). Compared with the scores in the control siRNA group (10.58 ± 0.36 and 12.50 ± 0.38), the neurological scores were improved in the siRNA group at 24 h and 72 h (12.67 ± 0.40, *p* = 0.001 and 15.00 ± 0.33, *p* = 0.000, respectively) (Figure 7B,C). RES treatment (12.50 ± 0.36, *p* = 0.016 and 14.83 ± 0.39, *p* = 0.002) increased the neurological scores compared with those of the NS group (11.16 ± 0.37 and 12.83 ± 0.44) at 24 h and 72 h (Figure 7B,C). However, there was no significant difference between the scores of the GSK2656157 (12.58 ± 0.36, *p* = 0.133 and 15.58 ± 0.31, *p* = 0.068) and DMSO (11.18 ± 0.32 and 14.58 ± 0.42, Figure 7B,C) control groups. These results suggested that TXNIP inhibition could attenuate the neurological deficits of SAH rats, but PERK inhibition showed no significant improvement.

### 2.7. BBB Permeability

Evans blue dye extravasation was used to measure the BBB permeability after SAH. In comparison to the sham group, SAH caused remarkable extravasation of Evans blue dye in the cerebral hemispheres, the cerebellum (CB), and the brain stem (BS) 72 h post-surgery (Figure 7D, *p* < 0.05). TXNIP siRNA injection significantly prevented BBB disruption in comparison to the control group, as reflected by the reduced amount of Evans blue dye in the left hemisphere (LH), right hemisphere (RH), and CB at 72 h (Figure 7D, *p* < 0.05). In addition, BBB disruption was also attenuated by RES injection in three brain regions (LH, RH, and CB) (Figure 7D, *p* < 0.05). Simultaneously, GSK2656157 treatment alleviated BBB disruption compared with the disruption observed in the DMSO control group (Figure 7D, *p* < 0.01). However, there were no significant differences in Evans blue dye extravasation in the BS between each group. These results showed that the inhibition of TXNIP and PERK could prevent BBB disruption in different regions after SAH.

### 2.8. Brain Water Content

SAH has been shown to cause marked brain edema in animal experiments, and diffuse brain swelling was also observed in this study at 72 h post-surgery. In comparison to the sham group, the brain water content was significantly deteriorated in the LH, RH, and CB at 72 h (Figure 7E and Table S1, *p* < 0.05). However, no significant differences in the BS were found between groups (Figure 7E, *p* = 0.310). SAH rats treated with RES exhibited lower brain water content in LH, RH, and CB when compared with rats in the NS group (Figure 7E and Table S1, *p* < 0.05). Additionally, the TXNIP siRNA treatment significantly reduced the water content in the LH, RH, and CB when compared with that in the control siRNA group (Figure 7E and Table S1, *p* < 0.001). The brain water content was also significantly reduced by GSK2656157 in comparison to the DMSO control group (Figure 7E and Table S1, *p* = 0.001). These results demonstrated that TXNIP and PERK inhibition could reduce cerebral edema after SAH.

## 3. Discussion

The present study proves that both pharmacological inhibition and gene knockdown of TXNIP significantly attenuated apoptosis and early prognostic symptoms after SAH. We found that these effects were closely associated with the TXNIP proapoptotic signalling pathway. For the first time, we found that TXNIP was extensively colocalized with TUNEL-positive cells in the rat hippocampus and subcortex after SAH. Meanwhile, PERK inhibition also showed significant TXNIP suppression and apoptosis attenuation. Together, our findings support the hypothesis that TXNIP may be a new effective target for EBI treatment after SAH.

Apoptosis is an important pathophysiological process of EBI that occurs rapidly in the SAH brain. Neurons, as well as astrocytes, oligodendrocytes, and vascular smooth muscle and endothelial cells, undergo apoptosis 24 h after SAH [20,21]. Various pro-apoptotic mechanisms are believed to be involved in EBI after SAH [1,21,22]. Caspase-dependent intrinsic pathway activation is considered to be the major factor that causes apoptosis, which not only includes the activation of proapoptotic proteins (BID, BAX, BAK, BAD, and BCL-XS), but also the inhibition of antiapoptotic proteins (BCL-2 and BCL-XL) [1,21,22].

Previous studies have reported that TXNIP is proapoptotic in ischaemic diseases [6,7,12]. However, whether or not TXNIP also influences the effect of SAH is still unknown. Kaya and colleagues reported that SAH significantly induced TXNIP mRNA expression [23]. Therefore, we speculated that TXNIP may also participate in EBI. TXNIP was first known as an endogenous inhibitor of cellular TRX that binds directly to TRX and exerts a proapoptotic function under pathologic conditions [24,25,26]. TRX1 is one of the TRX proteins that have various biological functions and can integrate with and inhibit ASK-1-dependent apoptosis [24]. TRX inhibition can induce neuronal apoptosis after cerebral ischaemia, which is extremely relevant to SAH-induced EBI [10,27]. Similarly, we found that TRX1 expression was inhibited after SAH (Figure 2), as TXNIP may colocalize with TRX1 and suppress TRX1 transcription [27]. Kim and colleges reported that TXNIP is proapoptotic and is located in neurons under ischaemic conditions [12]. In this study, we also found that TXNIP was widely expressed in neurons of the SAH rat brain (Figure 3A). More importantly, our TUNEL staining demonstrated for the first time that TXNIP was widely colocalized with apoptosis-positive cells both in the hippocampus and cortex after SAH (Figure 3B). These results may be the critical evidence necessary to further support our speculation. In the next experiment, when TXNIP expression was downregulated, cell apoptosis was significantly reduced, and the prognostic indicators of SAH were attenuated. Taken together, TXNIP may be associated with brain cell apoptosis in EBI after SAH. Although TXNIP is a promising therapeutic target, the application of TXNIP-specific inhibitors and agonists may be far more convincing in our study.

We previously found that sustainable PERK-C/EBP-homologous protein (CHOP) signalling activation could promote neuronal apoptosis after SAH [3,11]. As an important ER stress-related downstream signalling pathway, PERK-CHOP signalling is required at ER-mitochondrial contact sites to convey apoptosis [19,28]. Interestingly, irremediable ER stress can rapidly promote TXNIP upregulation [8,29]. Specifically, Oslowski and colleagues found that PERK significantly promoted TXNIP expression through ATF-5 and ChREBP transcription factors under ER stress [8]. The ER is the primary location for protein folding, posttranslational modification, and assembly [30]. Various stimuli, such as hypoxia and oxidative stress, can result in ER stress, which is a self-protective signal transduction pathway at first [31,32]. However, a high level of ER stress can culminate in cell death through promoting CHOP apoptosis signalling pathway activation [33,34]. Simultaneously, an increasing number of studies have shown that ER stress is implicated in the pathogenesis of cerebral ischaemia and SAH [3,35]. Our results found that PERK inhibition resulted in brain protection through the downregulation of TXNIP expression. Unfortunately, we did not detect the dose-dependent change of TXNIP and downstream apoptotic associated factors induced after GSK2656157 intervention, and we also did not measure the mRNA and immunofluorescence change of TXNIP after TXNIP siRNA and RES intervention, which would be more beneficial to confirm our conclusion. Meanwhile, as one of the limitations of the current study, we only tested the PERK phosphorylation levels. A more thorough method is to detect the total-PERK levels and then calculate the ratios between the p-PERK and total-PERK, which would provide more favourable evidence to support our results. Therefore, in a sense, these findings might indirectly suggest a potential link between ER stress, TXNIP, and SAH. Stress in the ER, as an important organelle in cells, might be a novel therapeutic target in SAH-related brain injury. These are shortcomings of this current study design and more work need to be done in our future work.

Activated PERK phosphorylates the eukaryotic translation initiation factor-2α (eIF-2α), inducing CHOP, ATF-5, and ChREBP expression [36]. In this study, PERK inhibition protected SAH-induced brain injury, but we could not exclude the possibility that some other mechanisms also exist. For example, Yan and colleagues found that PERK inhibition also exerts neuroprotective effects through Akt activation [37], which may explain why PERK inhibition did not attenuate the neurological deficits in our study. Meanwhile, other mechanisms also participate in the regulation of TXNIP expression and still deserve our further attention. Complementary to our results, Lerner and colleagues reported that hyperactivated inositol-requiring enzyme1α (IRE1α), a protein in the ER transmembrane like PERK, can also induce TXNIP expression at the posttranscriptional level through selective decay of TXNIP-destabilizing microRNA-17 [29]. More interestingly, TXNIP has been confirmed to participate in inflammatory amplification by activating the Nod-like receptor family, pyrin domain containing 3 (NLRP3) inflammasome [38]. Currently, both proinflammatory and proapoptotic factors have been demonstrated to be involved in SAH-induced brain injury [21]. In addition, ER stress can also occur in other pro-inflammatory immune or supporting cell types of the neurovascular unit, such as astrocytes, oligodendrocytes, vascular smooth muscle, and endothelial cells. It is also possible that ER-stress might induce pro-inflammatory activation of other cell types, which then can mediate indirect activation of TXNIP in neuronal cells in a paracrine matter. Finally, TXNIP possesses many kinds of biological functions and is important in multiple signalling pathways, which are worth researching thoroughly. Certainly, there are still many experiments that need to be done.

## 4. Materials and Methods

### 4.1. Animals

Two hundred fifty-nine adult male Sprague–Dawley rats (280–350 g) were obtained from the Animal Center of Chongqing Medical University. Animals were maintained in a specific pathogen-free laboratory with regular 12/12 h light/dark cycles under controlled temperature and humidity conditions. The Animal Ethics and Use Committee of Chongqing Medical University approved all experimental procedures (Permit No. SCXK (Chongqing) 2007-0001). All surgical procedures were performed under deep anaesthesia to minimize suffering, in accordance with the recommendation guidelines in the Care and Use of Laboratory Animals of the National Institutes of Health. Rats were divided into different groups: sham (*n* = 33), SAH (*n* = 63), SAH + control siRNA (Control, *n* = 23), SAH + TXNIP siRNA (*n* = 21), SAH + normal saline (NS, as a vehicle, *n* = 24), SAH + resveratrol (RES, *n* = 22), SAH + dimethyl sulfoxide (DMSO, as a vehicle, *n* = 28), and SAH + GSK2656157 (GSK, *n* = 45).

### 4.2. Endovascular Perforation Model of SAH

The endovascular perforation model is considered to be the most suitable model of SAH, given that there are many similarities in the pathophysiological changes observed with a clinical ruptured intracranial aneurysm [39]. SAH animal models were established through endovascular perforation as described before [40]. Rats were anaesthetized with an intraperitoneal injection of sodium pentobarbital (50 mg/kg), and supplemental doses (5 mg/kg) of pentobarbital were given to maintain anaesthesia when necessary. Body temperature was maintained at 37 °C using an electric heating pad during the operation. Sham-operated rats underwent identical procedures without the vessel puncture. After perforation, rats were kept in heated cages until recovery from anaesthesia.

### 4.3. Drug Administration

#### 4.3.1. Resveratrol and TXNIP siRNA Injection

Resveratrol (trans-3,4′,5-trihydroxystilbene, RES) was delivered to rats by intraperitoneal injection 1 h after puncture with a single dose of 60 mg/kg (1 mL, Sigma-Aldrich, St. Louis, MO, USA, −20 °C) [16]. RES, which is a natural phytoalexin in red wine, grape skin, and other plants with the ability to penetrate the BBB and rapidly reach brain tissue [14], has been reported to significantly suppress TXNIP mRNA and protein expression [7,15]. RES was dissolved in 50% ethanol and diluted with physiological saline. Normal saline (1 mL) containing 50% ethanol was used as the control.

Rats received TXNIP siRNA and control siRNA 24 h before the operation through intracerebroventricular infusion, as previously described [3]. Two different TXNIP siRNA were designed, as described before [17,18], and obtained from RiboBio Co., Ltd. (Guangzhou, China, Table 1). Briefly, 5 nmol siRNA mixture per rat in 6 μL of sterile phosphate-buffered saline (PBS) was inserted into the left lateral ventricle through a burr hole with a sterile 10-μL Hamilton syringe at a rate of 0.5 μL/min with the coordinates of 1.5 mm posterior, 1.0 mm lateral, and 3.2 mm below bregma in the horizontal plane [3]. Sham and SAH animals also received a burr hole, but no siRNA injection was performed. Ten minutes later, the needle was removed and the burr hole was carefully plugged with bone wax. 

#### 4.3.2. GSK2656157 Injection

GSK2656157 (MedChem Express, HY-13820, Shanghai, China) is an adenosine triphosphate (ATP)-competitive inhibitor that has been reported to effectively suppress PERK autophosphorylation through interaction with the PERK kinase domain [41]. However, the use of GSK2656157 in animal brain has not been reported. To avoid the necessity of the drug to cross the BBB and to improve the local drug concentration, SAH rats also received GSK2656157 through intracerebroventricular injection at the same coordinates as the TXNIP siRNA injection. According to a previous study, GSK2656157 can completely inhibit PERK autophosphorylation 8 h after treatment [41]. GSK2656157 was injected twice through two bilateral burr holes 8 h before and after surgery. Briefly, 10 μL of 1% DMSO in saline containing 30, 60, 120, 180, 240, or 300 μg of GSK2656157 were injected under deep anaesthesia with a sterile 10-μL Hamilton syringe at a rate of 0.5 μL/min. Ten minutes later, the needle was removed and the burr hole was plugged with bone wax. Rats in the control group received 10 μL of 1% DMSO in saline without the inhibitor. Under deep anaesthesia 24 h after surgery, the rats were quickly decapitated for western blot.

### 4.4. Neurological Scores

Neurological scores were evaluated at 24 and 72 h in a blinded fashion according to the scoring methods reported before [42]. An 18-point scoring system was used that consisted of six subtests, including spontaneous activity, spontaneous movements of four limbs, outstretching of the forelimbs, wall climbing of a wire cage, the trunk-touch reaction, and the vibrissae-touch response. Lower scores indicated serious neurological deficits.

### 4.5. SAH Grade and Mortality

After the evaluation of the neurological scores, rats were sacrificed to quantify the severity of SAH according to the previously described grading scale [42]. Each rat obtained a total score ranging from 0 to 18 after adding scores from all six subtests. Animals with a score below 5 points were ruled out due to their low SAH grade [42]. Meanwhile, mortality was recorded after the operation.

### 4.6. Blood-Brain Barrier (BBB) Permeability

BBB permeability assessment was based on Evans blue extravasation [43]. At 72 h after perforation, 4% Evans blue dye (2.5 mL/kg) was injected into the left femoral vein under deep anaesthesia and allowed to circulate for 60 min. Spectrophotometry was used to quantify the Evans blue dye at an excitation wavelength of 620 nm, an emission wavelength of 680 nm and a bandwidth of 10 nm.

### 4.7. Brain Water Content

Under deep anaesthesia, 72 h after perforation, the brains were quickly divided into the LH, RH, CB, and BS. These portions were weighed immediately (wet weight) and then kept in the oven for 72 h at 105 °C (dry weight). The brain water content percentage was recorded using the following equation: (wet weight to dry weight)/wet weight × 100% [3].

### 4.8. Immunofluorescence

Animals were sacrificed 24 h after perforation for immunofluorescence in accordance with our previous study [3]. Ten-micron-thick sections of SAH rat brains were incubated with primary antibodies against the following proteins (TXNIP (1:50, Abcam, Cambridge, MA, USA) and NEUN (1:100, Merck Millipore, Billerica, MA, USA) overnight at 4 °C. Fluorescence microscopy (FV1200, Olympus, Tokyo, Japan) was used to detect the localization of TXNIP in neurons. For negative controls, the same staining procedures were conducted without primary antibodies.

### 4.9. TUNEL Staining

Fluorescent TUNEL staining (Roche Inc., Mannheim, Germany) was performed according to the manufacturer’s protocol and a previous study [40]. TUNEL staining was used to detect TXNIP localization with apoptotic cells in the hippocampus and cortex of SAH animals by using fluorescence microscopy (IX71, Olympus, Japan). TUNEL-positive cells in the ipsilateral basal cortex (left) in five different fields per animal were counted under microscopy under 400× magnification in a blinded manner. The number of TUNEL-positive cells/mm^2^ predicted the severity of brain injury [44]. Meanwhile, negative controls were performed with labelling solution without the TUNEL reagent.

### 4.10. Western Blot

Under deep anaesthesia, rats were quickly decapitated, and the LHs were isolated for western blot, as described before [45]. Polyvinylidene fluoride (PVDF) membranes were incubated with primary antibodies against the following proteins: TXNIP (1:500; Abcam, Cambridge, MA, USA), TRX1 (1:2000; Abcam), ASK-1 (1:1000; Cell Signaling Technology, CST, Danvers, MA, USA), phosphorylated-ASK-1 (p-ASK-1,1:5000; Sigma-Aldrich, St. Louis, MO, USA), CC3 (1:1000; CST), BCL-2 (1:1000; CST), BAX (1:2000; Abcam), phosphorylated–PERK (p-PERK, 1:200; Santa Cruz Biotechnology, Santa Cruz, CA, USA), eIF2α (1:100; Santa Cruz), phosphorylated-eIF2α (p-eIF2α, 1:500; Abcam), ChREBP (1:100; Santa Cruz), ATF-5 (1:2000; Abcam), and β-actin (NeoBioscience Technology Co., Ltd., Beijing, China). Membranes were incubated with the appropriate horseradish peroxidase-conjugated secondary antibodies, visualized with the enhanced chemiluminescent reagent kit (ECL, Engreen Biosystem Co., Ltd., Beijing, China), and analysed with a Fusion gel imaging apparatus (Fusion fx 7 Spectra, Vilber, France). The results are expressed as a percentage of the values of β-actin.

### 4.11. Statistical Analysis

Statistical analysis was performed using SPSS17.0 software. The mortality data were analysed by Fisher’s exact test. All of the other data were expressed as the mean ± standard error of the mean, and t comparisons among multiple groups were performed by one-way analysis of variance (ANOVA), and followed by Dunnett’s *t*-test to compare between the control group and treatment group and the Student-Newman-Keuls test for between the two treatment groups with different intervention. *p* < 0.05 was considered to be statistically significant.

## 5. Conclusions

TXNIP may participate in EBI after SAH by mediating brain cell apoptosis. The blockage of TXNIP induced by PERK attenuated cell apoptosis and brain edema and improved the neurological score of SAH rats. Multiple independent proteins or interconnected signalling pathways may participate in the pathological process of EBI after SAH. These results suggest that TXNIP inhibition may be a potential therapeutic strategy after SAH. 

## Figures and Tables

**Table 1:** The sequences of the two different TXNIP siRNAs.

Name	Sequences
siRNA 1 [17]	sense: 5′-GCUGG AUAGACCUAAACAUTT-3′ antisense: 5′-AUGUUUAGGUCUAUCCAGCTT-3′
siRNA 2 [18]	sense: 5′-UGGUCACGUCGAAAUGAAUTT-3′ antisense: 5′-TTACCAGUGCAGCUUUACUUA-3′
Control siRNA	sense: 5′-UUCUCCGAACGUGUCACGUTT-3′ antisense: 5′-ACGUGACACGUUCGGAGAATT-3′

## Supplementary Materials

Supplementary materials can be found at www.mdpi.com/1422-0067/18/4/854/s1.

## References

[1] Cahill J., Calvert J.W., Zhang J.H. (2006). Mechanisms of early brain injury after subarachnoid hemorrhage. J. Cereb. Blood Flow Metab..

[2] Hasegawa Y., Suzuki H., Sozen T., Altay O., Zhang J.H. (2011). Apoptotic mechanisms for neuronal cells in early brain injury after subarachnoid hemorrhage. Acta Neurochir. Suppl..

[3] He Z., Ostrowski R.P., Sun X., Ma Q., Huang B., Zhan Y., Zhang J.H. (2012). Chop silencing reduces acute brain injury in the rat model of subarachnoid hemorrhage. Stroke J. Cereb. Circ..

[4] Alvarez C.E. (2008). On the origins of arrestin and rhodopsin. BMC Evol. Biol..

[5] Saxena G., Chen J., Shalev A. (2010). Intracellular shuttling and mitochondrial function of thioredoxin-interacting protein. J. Biol. Chem..

[6] Su H., Ji L., Xing W., Zhang W., Zhou H., Qian X., Wang X., Gao F., Sun X., Zhang H. (2013). Acute hyperglycaemia enhances oxidative stress and aggravates myocardial ischaemia/reperfusion injury: Role of thioredoxin-interacting protein. J. Cell. Mol. Med..

[7] Ishrat T., Mohamed I.N., Pillai B., Soliman S., Fouda A.Y., Ergul A., El-Remessy A.B., Fagan S.C. (2015). Thioredoxin-interacting protein: A novel target for neuroprotection in experimental thromboembolic stroke in mice. Mol. Neurobiol..

[8] Oslowski C.M., Hara T., O’Sullivan-Murphy B., Kanekura K., Lu S., Hara M., Ishigaki S., Zhu L.J., Hayashi E., Hui S.T. (2012). Thioredoxin-interacting protein mediates er stress-induced β cell death through initiation of the inflammasome. Cell Metab..

[9] Czabotar P.E., Lessene G., Strasser A., Adams J.M. (2014). Control of apoptosis by the bcl-2 protein family: Implications for physiology and therapy. Nat. Rev. Mol. Cell Biol..

[10] Wu X., Li L., Zhang L., Wu J., Zhou Y., Zhou Y., Zhao Y., Zhao J. (2015). Inhibition of thioredoxin-1 with sirna exacerbates apoptosis by activating the ask1-jnk/p38 pathway in brain of a stroke model rats. Brain Res..

[11] He Z., Ostrowski R.P., Sun X., Ma Q., Tang J., Zhang J.H. (2012). Targeting c/ebp homologous protein with sirna attenuates cerebral vasospasm after experimental subarachnoid hemorrhage. Exp. Neurol..

[12] Kim G.S., Jung J.E., Narasimhan P., Sakata H., Chan P.H. (2012). Induction of thioredoxin-interacting protein is mediated by oxidative stress, calcium, and glucose after brain injury in mice. Neurobiol. Dis..

[13] Negoescu A., Lorimier P., Labat-Moleur F., Drouet C., Robert C., Guillermet C., Brambilla C., Brambilla E. (1996). In situ apoptotic cell labeling by the tunel method: Improvement and evaluation on cell preparations. J. Histochem. Cytochem..

[14] Baur J.A., Sinclair D.A. (2006). Therapeutic potential of resveratrol: The in vivo evidence. Nat. Rev. Drug Dis..

[15] Bedarida T., Baron S., Vibert F., Ayer A., Henrion D., Thioulouse E., Marchiol C., Beaudeux J.L., Cottart C.H., Nivet-Antoine V. (2016). Resveratrol decreases txnip mrna and protein nuclear expressions with an arterial function improvement in old mice. J. Gerontol. Ser. A Biol. Sci. Med. Sci..

[16] Zhou X.M., Zhou M.L., Zhang X.S., Zhuang Z., Li T., Shi J.X., Zhang X. (2014). Resveratrol prevents neuronal apoptosis in an early brain injury model. J. Surg. Res..

[17] Xiao J., Zhu Y., Liu Y., Tipoe G.L., Xing F., So K.-F. (2014). Lycium barbarum polysaccharide attenuates alcoholic cellular injury through txnip-nlrp3 inflammasome pathway. Int. J. Biol. Macromol..

[18] Luo B., Li B., Wang W., Liu X., Xia Y., Zhang C., Zhang M., Zhang Y., An F. (2014). Nlrp3 gene silencing ameliorates diabetic cardiomyopathy in a type 2 diabetes rat model. PLoS ONE.

[19] Verfaillie T., Rubio N., Garg A.D., Bultynck G., Rizzuto R., Decuypere J.P., Piette J., Linehan C., Gupta S., Samali A. (2012). Perk is required at the er-mitochondrial contact sites to convey apoptosis after ros-based er stress. Cell Death Differ..

[20] Park S., Yamaguchi M., Zhou C., Calvert J.W., Tang J., Zhang J.H. (2004). Neurovascular protection reduces early brain injury after subarachnoid hemorrhage. Stroke J. Cereb. Circ..

[21] Sehba F.A., Hou J., Pluta R.M., Zhang J.H. (2012). The importance of early brain injury after subarachnoid hemorrhage. Prog. Neurobiol..

[22] Broughton B.R., Reutens D.C., Sobey C.G. (2009). Apoptotic mechanisms after cerebral ischemia. Stroke J. Cereb. Circ..

[23] Kaya B., Erdi F., Kilinc I., Keskin F., Feyzioglu B., Esen H., Karatas Y., Uyar M., Kalkan E. (2015). Alterations of the thioredoxin system during subarachnoid hemorrhage-induced cerebral vasospasm. Acta Neurochir..

[24] Saitoh M., Nishitoh H., Fujii M., Takeda K., Tobiume K., Sawada Y., Kawabata M., Miyazono K., Ichijo H. (1998). Mammalian thioredoxin is a direct inhibitor of apoptosis signal-regulating kinase (ask) 1. EMBO J..

[25] Nishiyama A., Matsui M., Iwata S., Hirota K., Masutani H., Nakamura H., Takagi Y., Sono H., Gon Y., Yodoi J. (1999). Identification of thioredoxin-binding protein-2/vitamin d3 up-regulated protein 1 as a negative regulator of thioredoxin function and expression. J. Biol. Chem..

[26] Hwang J., Suh H.W., Jeon Y.H., Hwang E., Nguyen L.T., Yeom J., Lee S.G., Lee C., Kim K.J., Kang B.S. (2014). The structural basis for the negative regulation of thioredoxin by thioredoxin-interacting protein. Nat. Commun..

[27] Yoshihara E., Masaki S., Matsuo Y., Chen Z., Tian H., Yodoi J. (2014). Thioredoxin/txnip: Redoxisome, as a redox switch for the pathogenesis of diseases. Front. Immunol..

[28] Tabas I., Ron D. (2011). Integrating the mechanisms of apoptosis induced by endoplasmic reticulum stress. Nat. Cell Biol..

[29] Lerner A.G., Upton J.-P., Praveen P., Ghosh R., Nakagawa Y., Igbaria A., Shen S., Nguyen V., Backes B.J., Heiman M. (2012). Ire1α induces thioredoxin-interacting protein to activate the nlrp3 inflammasome and promote programmed cell death under irremediable er stress. Cell Metab..

[30] Roussel B.D., Kruppa A.J., Miranda E., Crowther D.C., Lomas D.A., Marciniak S.J. (2013). Endoplasmic reticulum dysfunction in neurological disease. Lancet. Neurol..

[31] Ron D., Walter P. (2007). Signal integration in the endoplasmic reticulum unfolded protein response. Nat. Rev. Mol. Cell Biol..

[32] Walter P., Ron D. (2011). The unfolded protein response: From stress pathway to homeostatic regulation. Science.

[33] Szegezdi E., Logue S.E., Gorman A.M., Samali A. (2006). Mediators of endoplasmic reticulum stress-induced apoptosis. EMBO Rep..

[34] Smith M.I., Deshmukh M. (2007). Endoplasmic reticulum stress-induced apoptosis requires bax for commitment and apaf-1 for execution in primary neurons. Cell Death Differ..

[35] Tajiri S., Oyadomari S., Yano S., Morioka M., Gotoh T., Hamada J.I., Ushio Y., Mori M. (2004). Ischemia-induced neuronal cell death is mediated by the endoplasmic reticulum stress pathway involving chop. Cell Death Differ..

[36] Schroder M., Kaufman R.J. (2005). The mammalian unfolded protein response. Annu. Rev. Biochem..

[37] Yan F., Cao S., Li J., Dixon B., Yu X., Chen J., Gu C., Lin W., Chen G. (2016). Pharmacological inhibition of perk attenuates early brain injury after subarachnoid hemorrhage in rats through the activation of akt. Mol. Neurobiol..

[38] Zhou R., Tardivel A., Thorens B., Choi I., Tschopp J. (2010). Thioredoxin-interacting protein links oxidative stress to inflammasome activation. Nat. Immunol..

[39] Lee J.Y., Sagher O., Keep R., Hua Y., Xi G. (2009). Comparison of experimental rat models of early brain injury after subarachnoid hemorrhage. Neurosurgery.

[40] Duris K., Manaenko A., Suzuki H., Rolland W.B., Krafft P.R., Zhang J.H. (2011). A7 nicotinic acetylcholine receptor agonist pnu-282987 attenuates early brain injury in a perforation model of subarachnoid hemorrhage in rats. Stroke J. Cereb. Circ..

[41] Atkins C., Liu Q., Minthorn E., Zhang S.Y., Figueroa D.J., Moss K., Stanley T.B., Sanders B., Goetz A., Gaul N. (2013). Characterization of a novel perk kinase inhibitor with antitumor and antiangiogenic activity. Cancer Res..

[42] Sugawara T., Ayer R., Jadhav V., Zhang J.H. (2008). A new grading system evaluating bleeding scale in filament perforation subarachnoid hemorrhage rat model. J. Neurosci. Methods.

[43] Tsubokawa T., Solaroglu I., Yatsushige H., Cahill J., Yata K., Zhang J.H. (2006). Cathepsin and calpain inhibitor e64d attenuates matrix metalloproteinase-9 activity after focal cerebral ischemia in rats. Stroke J. Cereb. Circ..

[44] Hasegawa Y., Suzuki H., Altay O., Zhang J.H. (2011). Preservation of tropomyosin-related kinase b (trkb) signaling by sodium orthovanadate attenuates early brain injury after subarachnoid hemorrhage in rats. Stroke J. Cereb. Circ..

[45] Suzuki H., Hasegawa Y., Kanamaru K., Zhang J.H. (2010). Mechanisms of osteopontin-induced stabilization of blood-brain barrier disruption after subarachnoid hemorrhage in rats. Stroke J. Cereb. Circ..

