# Supplementary material for: Thioredoxin-Interacting Protein Mediates Apoptosis in Early Brain Injury after Subarachnoid Haemorrhage
Source: Int J Mol Sci. 2017 Apr 18;18(4):854. doi: 10.3390/ijms18040854 (PMC5412438; doi:10.3390/ijms18040854)
Supplement: Supplementary file 1 [file ijms-18-00854-s001.pdf]

Supplementary Materials: Thioredoxin-Interacting Protein Mediates Apoptosis in Early Brain Injury after Subarachnoid Haemorrhage

Qing Zhao, Xudong Che, Hongxia Zhang, Guanping Tan, Liu Liu, Dengzhi Jiang, Jun Zhao, Xiang Xiang, Xiaochuan Sun and Zhaohui He

Table S1. Brain water content.

Groups	LH	RH	CB	BS
Sham	78.45 ± 0.09%	78.41 ± 0.06%	78.31 ± 0.05%	72.74 ± 0.20%
SAH	78.98 ± 0.15%	78.87 ± 0.40%	78.78 ± 0.17%	73.06 ± 0.28%
Control siRNA	78.92 ± 0.31%	79.12 ± 0.24%	78.81 ± 0.24%	72.97 ± 0.15%
TXNIP siRNA	78.66 ± 0.17%	78.73 ± 0.11%	78.55 ± 0.12%	72.89 ± 0.17%
Normal saline	78.96± 0.08%	78.94 ± 0.07%	78.93± 0.10%	72.50 ± 0.20%
RES	78.63 ± 0.09%	78.64 ± 0.13%	78.68 ± 0.12%	72.69 ± 0.12%
DMSO	79.23 ± 0.20%	79.11 ± 0.24%	78.96 ± 0.09%	72.28 ± 0.12%
GSK2656157	78.61 ± 0.12%	78.75 ± 0.17%	78.67 ± 0.08%	72.48 ± 0.17%

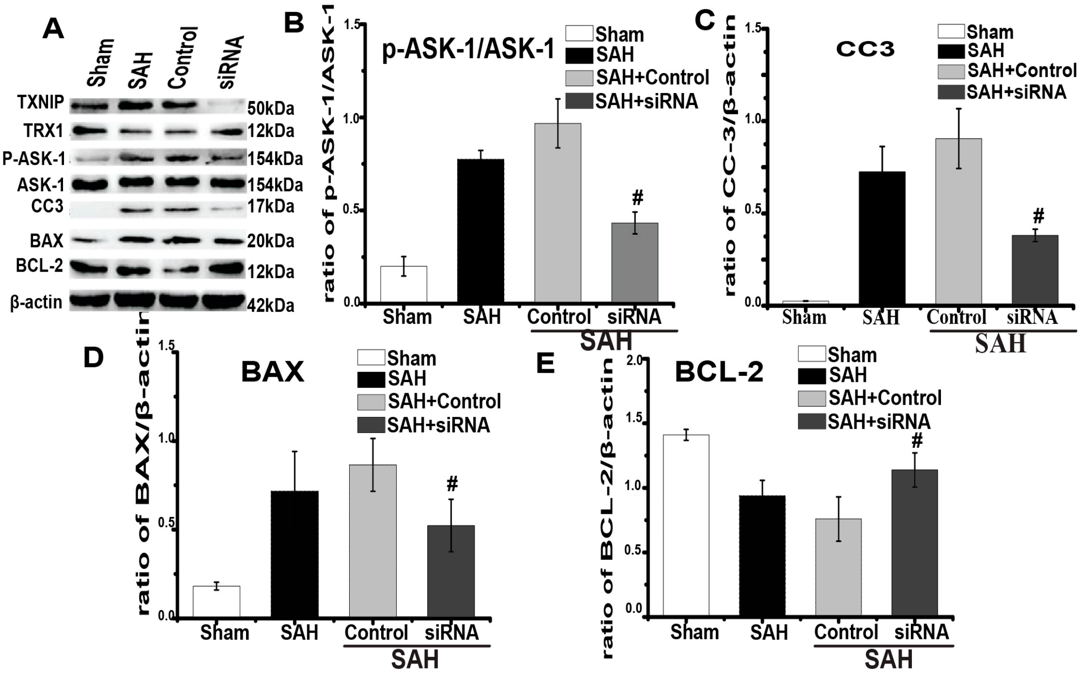

Figure S1. P-ASK-1, ASK-1, cleaved-Caspase-3, BAX and BCL-2 expression after TXNIP siRNA treatment. (**A–E**) Representative Western blot of TXNIP, TRX1, p-ASK-1/ASK-1, cleaved Caspase-3, BAX and BCL-2. P-ASK-1/ASK-1, cleaved Caspase-3, BAX and BCL-2 expression were downregulated by TXNIP siRNA. # $p < 0.05$ vs. SAH + NS.

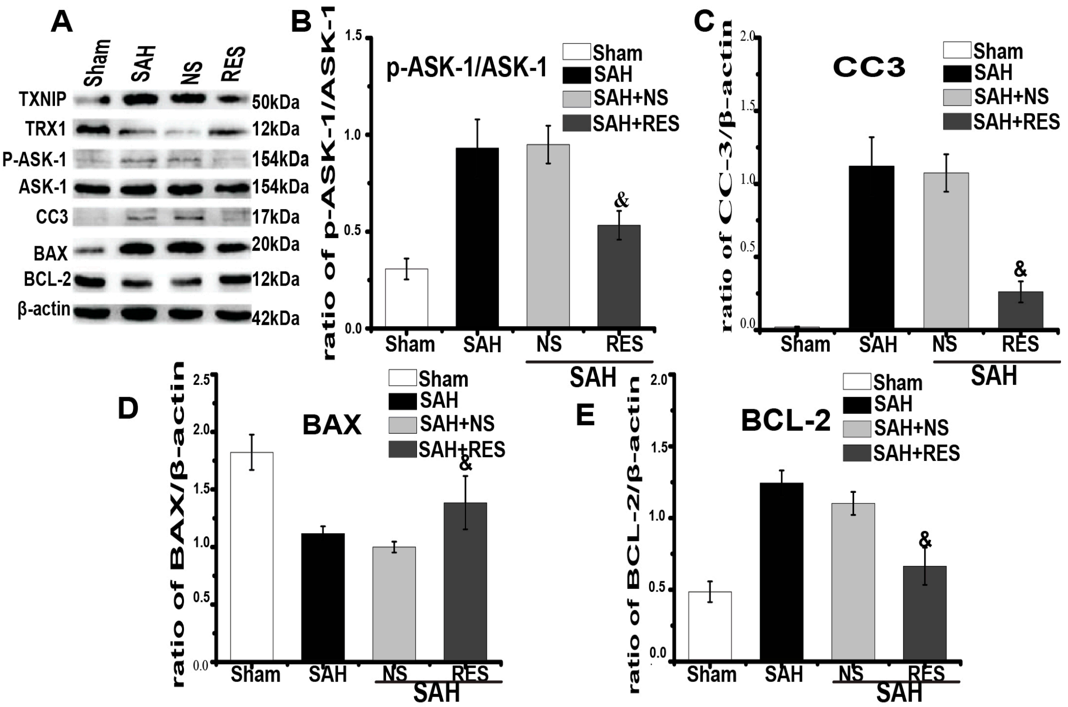

Figure S2. P-ASK-1, ASK-1, cleaved-Caspase-3, BAX and BCL-2 expression after Resveratrol (RES) treatment. (**A**–**E**) Representative Western blot of TXNIP, TRX1, p-ASK-1/ASK-1, cleaved Caspase-3, BAX and BCL-2. P-ASK-1/ASK-1, cleaved Caspase-3, BAX and BCL-2 expression were downregulated by Resveratrol (RES). & $p < 0.05$ vs. SAH + NS.

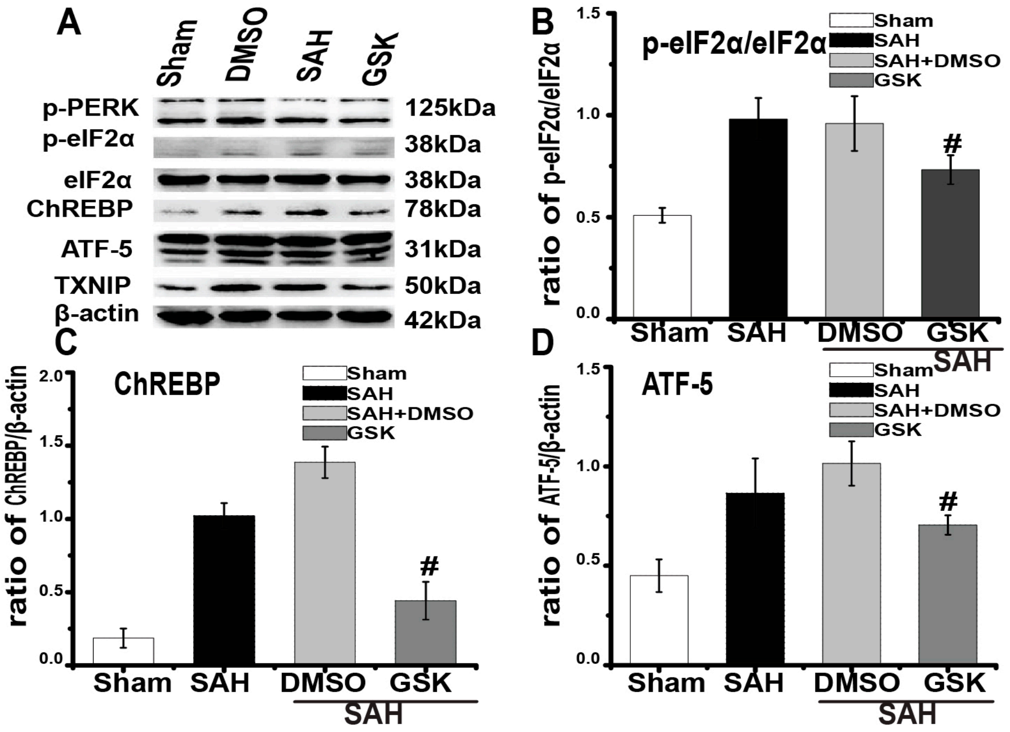

Figure S3. GSK2656157 treatment suppressed the expression of PERK downstream proteins. (**A**–**D**) Representative Western blot of p-PERK, TXNIP, p-eIF2α/eIF2α, ATF-5 and ChREBP. PERK downstream proteins expression were downregulated by GSK2656157 treatment. # $p < 0.05$ vs. SAH + DMSO.
